# Simultaneous occurrence of papillary thyroid carcinoma, medullary thyroid carcinoma, and lymphoma: A case report

**DOI:** 10.1097/MD.0000000000039363

**Published:** 2024-08-16

**Authors:** Zengfang Hao, Hanjing Cui, Yuehong Li, Wenxin Wu, Yuan Wang, Haijun Dan, Lei Lou, Hengshu Wang, Pengxin Zhao

**Affiliations:** a Department of Pathology, The Second Hospital of Hebei Medical University, Shijiazhuang City, Hebei Province, China; b Department of Thyroid and Breast Surgery, The Second Hospital of Hebei Medical University, Shijiazhuang, Hebei, China; c Department of Ultrasonography, The Second Hospital of Hebei Medical University, Shijiazhuang, Hebei, China.

**Keywords:** collision tumor, medullary thyroid carcinoma (PTC), papillary thyroid carcinoma (MTC), small lymphocytic lymphoma (SLL)

## Abstract

**Background:**

Papillary thyroid carcinoma (PTC) is the most common type of thyroid cancer, the coexistence of PTC and medullary thyroid carcinoma (MTC) is uncommon. While the simultaneous occurrence of both cancers with small lymphocytic lymphoma (SLL) in lymph nodes with PTC metastasis is very rare. This study presents a unique case of concurrent PTC, MTC, and SLL, highlighting the exceptional rarity of these coexisting tumors.

**Methods:**

A 75-year-old female with a thyroid tumor underwent total thyroidectomy, bilateral central neck lymph node dissection, and right radical neck lymph node dissection. Histopathological examination revealed a low-grade medullary thyroid carcinoma (MTC) in the left lobe and classical papillary thyroid carcinoma (PTC) in the right lobe, with PTC metastasis in the cervical lymph nodes and concurrent SLL in the affected lymph nodes.

**Results:**

Coexistence of PTC, MTC and SLL in the same patient is rare, there are currently no standardized treatment guidelines due to the limited literature. However, it is essential to consider not only the treatment for each type of tumor but also the potential risks or conflicts associated with the treatments. In the case reported in this paper, the papillary carcinoma invaded the capsule of the right lobe of the thyroid and metastasized to the cervical lymph nodes, warranting radioactive iodine therapy. However, considering the potential negative impact of radioactive iodine on the pre-existing lymphoma, the radioactive iodine therapy was postponed. Meanwhile, constant monitoring of calcitonin and thyroid globulin should be performed to monitor tumor recurrence as was performed in the present case.

**Conclusion:**

Since MTC, PTC, and SLL may coexist, patients with PTC deserve careful surveillance for the other disease entities. This case underscores the need for heightened awareness among clinicians, radiologists, and pathologists regarding the possibility of concurrent thyroid tumors and abnormal lymph nodes, guiding comprehensive pre-operative evaluations and postoperative monitoring strategies. This study aims to provide a warning for routine pathological diagnosis and contribute data for related research.

## 1. Introduction

The majority of primary malignant tumors of the thyroid gland arise from the follicular epithelium, with over 90% being differentiated thyroid carcinomas, including papillary thyroid carcinoma (PTC) and follicular carcinomas; PTC comprises the vast majority of thyroid differentiated thyroid carcinomas cases (85%).^[1,2]^ The incidence of PTC has significantly increased in recent decades, mainly owing to advancements in diagnostic methods.^[2]^ Medullary thyroid carcinoma (MTC) is a rare type of primary thyroid tumor that is much less common than PTC, accounting for 3% to 5% of all thyroid cancers. The MTC originates from parafollicular cells (C cells) within the thyroid and is derived from the neural crest; therefore, it is considered a neuroendocrine tumor.^[3,4]^ Chronic lymphocytic leukemia/small lymphocytic lymphoma (CLL/SLL) is characterized by the clonal proliferation and accumulation of mature B cells, typically CD5-positive, in the blood, bone marrow, lymph nodes, and spleen. It predominantly affects middle-aged to older individuals and represents an indolent lymphoproliferative disorder of the hematopoietic system.^[5]^ SLL refers to cases with tissue morphology and immunophenotype of CLL but without leukemia manifestation.

Patients with the coexistence of 2 malignancies, such as PTC with MTC^[6]^ and PTC with SLL,^[7]^ have been reported. While synchronous occurrence of PTC, MTC, and SLL is rare in the literature,^[8]^ we report a case of a 75-year-old female patient with concurrent of PTC and MTC in different lobes of the thyroid. Additionally, this patient had lymphoma (SLL) involvement and PTC metastasis in the same lymph node. These three tumors originate from different tissues or cells and exhibit distinct clinical manifestations and biological behaviors. The treatment strategy and prognosis differ significantly, necessitating accurate diagnosis of these co-existing tumors.

## 2. Case presentation

An older female patient aged 75 years presented to the hospital complaining of neck discomfort and pain for the past month without polydipsia, polyphagia, polyuria, asthenia, weight loss, hoarseness, dysphagia, dyspnea, fever, diaphoresis, or hepatosplenomegaly. The patient sought consultation and further treatment at the Endocrine Surgery Outpatient Clinic of our hospital.

Physical examination revealed symmetrical neck on both sides; a nodular lesion, approximately 3 × 2.5 cm in size, palpable on the right lobe of the thyroid gland; the nodule was firm with indistinct borders and an irregular surface; mobility of the nodule was limited; no palpable cervical lymphadenopathy was not observed on either side of the neck or along the supraclavicular regions.

Past Medical History: Over 30 years ago, left mastectomy was performed locally without pathological examination owing to limited conditions at the primary hospital; therefore, no further detailed information is available, no history of hypertension, coronary artery disease, diabetes mellitus, hepatitis, tuberculosis, typhoid disease, or other illnesses. Systematic review did not reveal any specific issues, no history of radiotherapy or chemotherapy. The patient had a history of allergy to cephalosporin antibiotics.

**Figure 1.**
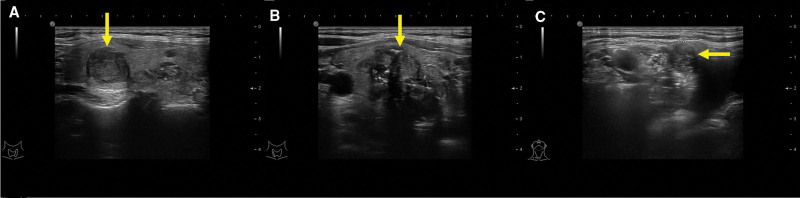
(A) Irregular solid nodule in the left lobe of the thyroid (arrow). (B) Irregularly shaped, poorly defined solid nodule in the right lobe of the thyroid (arrow). (C) Enlarged lymph nodes with indistinct corticomedullary differentiation (arrow).

Thyroid ultrasound revealed a hypoechoic irregular nodule measuring approximately 1.4 × 1.3 cm with a strong echo and rich blood flow signal in the upper pole of the left thyroid lobe, RI: 0.89. In the lower pole of the right thyroid lobe, a hypoechoic irregular nodule measuring about 3.3 × 2.5 cm with a strong echo, posterior shadowing, and band-like blood flow signal was identified, RI: 0.80. A hypoechoic lymph node measuring about 1.1 × 1.0 cm with band-like blood flow signal was observed in the right neck level IV region (Fig. 1).

Laboratory tests revealed elevated serum thyroglobulin (TG) levels (330 ng/mL), increased thyroid-stimulating hormone (TSH) levels (12.63 uIU/mL), elevated serum calcitonin (cal) levels (41.5 pmol/L), lymphocyte percentage of 53.7%, lymphocyte absolute value of 3.10 × 10^9^/L, red blood cell count of 4.13 × 10^12^/L, and hemoglobin level of 114 g/L.

Owing to the patient’s refusal to undergo fine-needle aspiration, we decided to excise the thyroid nodules for intraoperative frozen section pathological examination to clarify the nature of the lesion. After extensive discussions among multiple physicians and confirmation that there were no surgical contraindications, the patient underwent intraoperative frozen section pathology of the bilateral thyroid nodules, which revealed MTC in the left lobe and PTC in the right lobe. Subsequently, the patient underwent a total thyroidectomy, bilateral central neck lymph node dissection, and right radical neck lymph node dissection. During surgery, a solid hard nodule measuring approximately 1.4 cm × 1.3 cm × 1 cm with unclear borders was found in the upper pole of the left thyroid lobe, while a 3.3 cm × 2.5 cm × 2 cm solid hard nodule invading the capsule was identified in the lower part of the right lobe.

**Figure 2.**
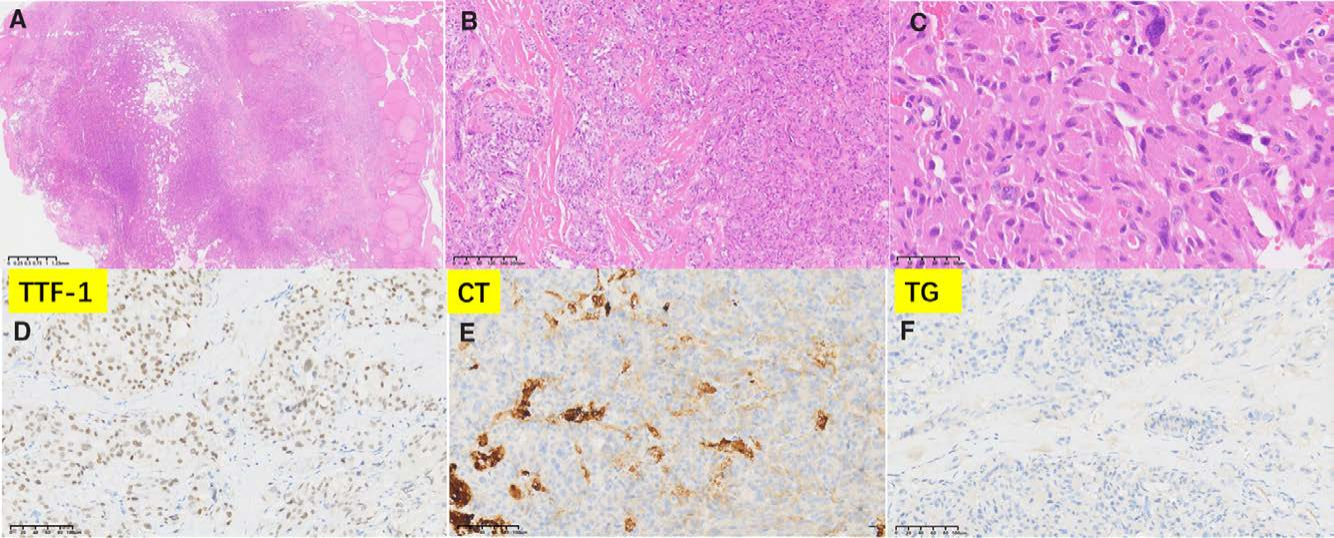
(A–C) Scanner view displaying the medullary carcinoma region in the left lobe of the thyroid. (D–F) Immunohistochemical staining showing that the tumor cells are positive for TTF-1 and CT but negative for TG. CT = calcitonin, TG = thyroglobulin, TTF-1 = thyroid transcription factor-1.

**Figure 3.**
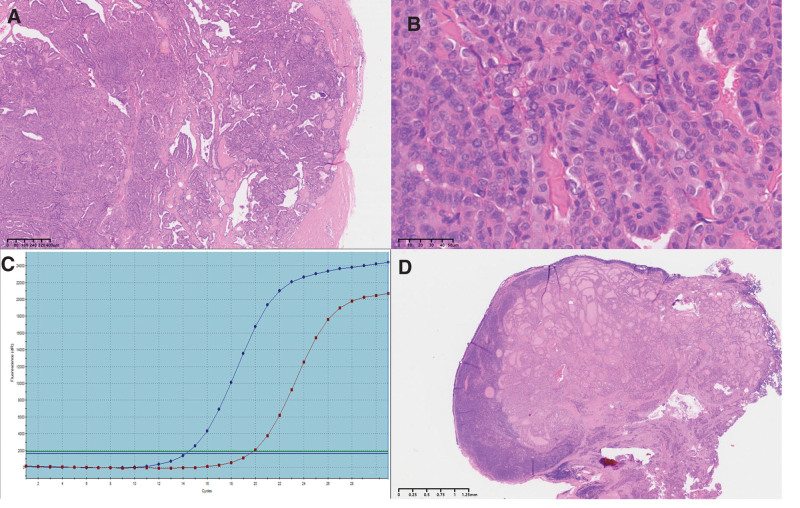
(A, B) The nodule in the right lobe of the thyroid is consistent with classical papillary thyroid carcinoma. (C) The tumor tissue has a BRAF V600E gene mutation. (D) Simultaneous presence of metastatic carcinoma and SLL within the lymph node. SLL = small lymphocytic lymphoma.

Histopathological examination revealed clear boundaries between the nodule in the left lobe and the surrounding thyroid tissue, with focal infiltration around the thyroid tissue. The tumor cells exhibited nests and trabecular growth patterns of varying sizes, with focal follicular structures and amyloid deposits in the stroma. The tumor cells were round or polygonal in shape, with abundant eosinophilic cytoplasm, oval-to spindle-shaped nuclei, coarse granular chromatin, giant cells, and no evidence of necrosis or nuclear division. Immunohistochemical staining showed positive results for thyroid transcription factor-1, calcitonin, CgA, and Ki-67 (3%) and negative results for TG (Fig. 2). According to the diagnostic criteria of the 2022 WHO classification of thyroid tumors, the nodule in the left lobe was diagnosed as a low-grade MTC and the nodule in the right lobe as classical PTC, presenting with complex branching papillary and follicular structures, enlarged and crowded cells, oval nuclei with pale dust-like chromatin, irregular nuclear membranes, visible nuclear grooves, intranuclear pseudoinclusions, and a BRAF V600E mutation. Metastasis of papillary carcinoma was observed in the central and right cervical lymph nodes (Fig. 3).

**Figure 4.**
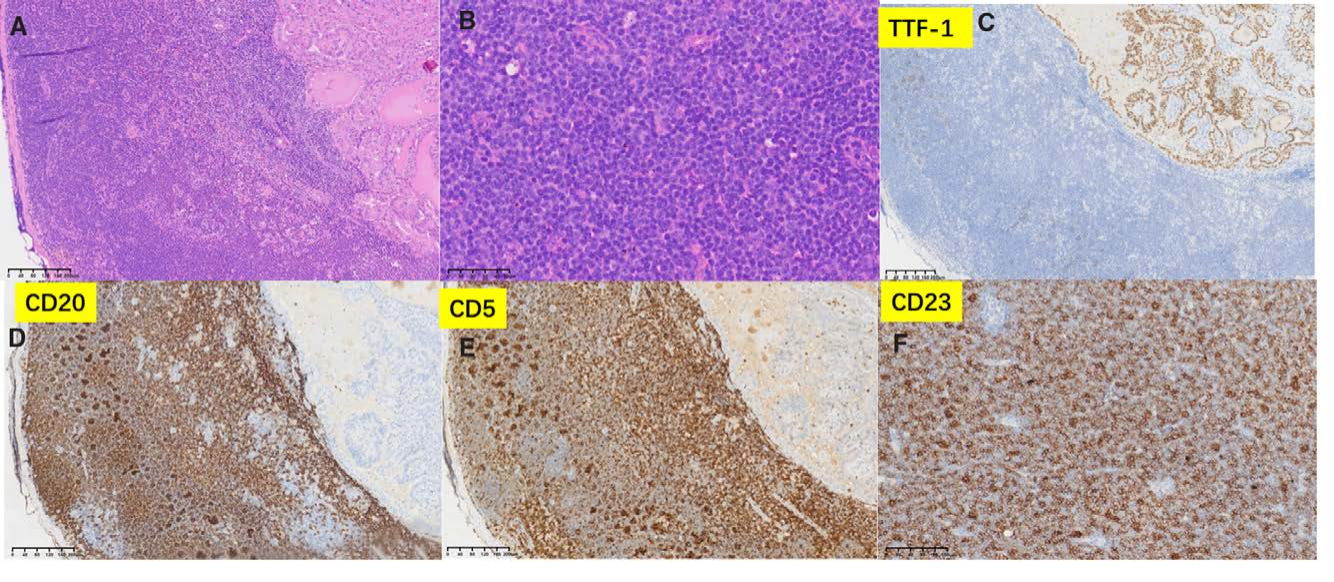
(A) The upper right part exhibits metastatic carcinoma, while the lower left part shows SLL. (B) SLL presents as diffuse sheets of small lymphocytic-like cells. (C) The metastatic carcinoma area is positive for thyroid transcription factor-1 (TTF-1). (D–F) The SLL region is positive for CD20, CD5, and CD23. SLL = small lymphocytic lymphoma.

Enlarged lymph nodes in the neck revealed PTC metastasis; loss of normal lymphoid tissue architecture; diffuse growth of small lymphocyte-like cells with scant cytoplasm; round deeply stained nuclei; rare nuclear divisions; scattered pale areas forming pseudofollicles (proliferation centers); immunohistochemical staining positive for CD20, PAX-5, CD5, CD23, and Ki-67 (10%) and negative for CD3, CD21, CD10, and cyclin D1 (Fig. 4).

The patient was ultimately diagnosed with low-grade medullary carcinoma in the left thyroid lobe, papillary carcinoma in the right thyroid lobe with metastasis to the cervical lymph nodes, and an SLL in the cervical lymph nodes. The pathological staging of MTC and PTC in the left and right thyroid lobes was PT1bN0M0 and PT2N1M0, respectively. Two weeks postsurgery, the patient underwent whole-body PET-CT scan, which confirmed no metastasis to other sites. Following total thyroidectomy, the patient was prescribed Levothyroxine 100 μg/day orally and scheduled for regular follow-up neck ultrasound, serum levels of TSH, FT3, FT4, TG, and calcitonin (CT) levels; no other medication was administered. Six months postsurgery, a follow-up investigation was conducted on the patient. Repeat neck ultrasound, serum thyroglobulin, and calcitonin tests showed no abnormalities. The patient sought consultation with a hematologist regarding a subsequent treatment plan for the SLL. Following a thorough evaluation by the physician, treatment for small lymphocytic lymphoma can be deferred for now, considering the indolent nature of the disease and based on the clinical presentation and other relevant examinations.

## 3. Discussion

Herein, we report, for the first time, an extremely rare case of coexistence of PTC, MTC, and SLL. This case underscores the need for special caution in diagnosing patients with multiple cancers, emphasizing the importance of establishing an effective testing system to determine the origin of coexisting tumors. Moreover, there is a critical need to develop effective treatments for patients with multiple tumors.

In clinical practice, concurrent diagnoses of PTC and MTC are exceedingly rare, accounting for <1% of thyroid tumors.^[6,9,10]^ The presentation pattern can be of 2 types: mixed medullary/follicular thyroid carcinoma, in which the 2 components are present within the same neoplastic nodule, or PTC/MTC, in which the 2 components are separated from the healthy thyroid tissue.^[11]^ Our case showed the PTC/MTC mode, and the nodule in the left lobe was diagnosed as low-grade MTC, while that in the right lobe was identified as classical PTC.

The pathogenic mechanisms underlying thyroid collision tumors remain unclear, but several hypotheses have been proposed. The tumor-induced tumor theory suggests that 1 tumor triggers changes in the organ microenvironment that lead to the development of a second tumor. Common stem cell origin theories propose that tumors arise from stem cells capable of differentiating into various tumor cell types or from 2 distinct driving mutations in a common stem cell that result in separate tumors. Stochastic collisional effect theory posits that the 2 tumors have independent origins, and their simultaneous occurrence is merely coincidental.^[12–15]^ To date, it has been excluded that the classic mutations involving RET, BRAF, and RAS oncogenes found in cases of exclusive PTC and MTC could be the basis of PTC/MTC collision tumors.^[16]^

Concomitant occurrence of thyroid carcinoma and lymphoma in the same patient is extremely rare. Individual cases have reported the concurrence of PTC with Hodgkin’s lymphoma, mucosa-associated lymphoid tissue lymphoma, diffuse large B-cell lymphoma, anaplastic large cell lymphoma, and SLL.^[17–21]^ However, there have been no reported cases of the simultaneous occurrence of MTC and lymphoma. Sezer et al^[7]^ reported a case involving a patient with a history of CLL, where CLL/SLL involvement and PTC metastasis in the same lymph node were reported. In contrast, in the present case, SLL was diagnosed upon lymph node removal during the search for cancer metastasis. SLL specifically affected the cervical lymph nodes, and there was no presentation of CLL in this patient.

In addition to the concurrence of PTC and MTC, as well as PTC and SLL, the most distinctiveness of this individual case is the concurrence of these 3 tumors (PTC, MTC, and SLL), which has been reported in only 1 similar case,^[8]^ which differs from the case reported in this article in 2 aspects: first, lymph nodes with lymphoma in the previous literature did not exhibit cancer metastasis; second, the patients in the previous literature had a history of radiation therapy, which may have been associated with the occurrence of lymphoma in those patients, while the patient in this study had no history of radiation therapy or exposure to radioactive materials.

Owing to the limited literature on the coexistence of PTC and MTC, there are currently no standardized treatment guidelines. Some researchers have suggested that PTC and MTC should be treated as separate autonomous tumors. Conversely, other scholars argue that the treatment approach should be determined by the most aggressive tumor, typically involving surgical resection and adjuvant therapy.^[13,14]^ If the PTC component exhibits high-risk characteristics, adjuvant radioactive iodine therapy should be considered. Biscola et al reported that the presence of PTC and subsequent radioactive iodine therapy did not affect the outcome of the MTC component in coexisting tumors. The prognosis of the coexistence of PTC and MTC appears to correlate with the staging of more aggressive MTC. A consensus has been reached that in cases of concurrent MTC and PTC, constant monitoring of calcitonin and thyroid globulin levels should be performed to monitor tumor recurrence. Interestingly, the prognosis of patients with coexisting MTC and PTC appears to be better than that of MTC alone.^[22]^ In the case reported in this paper, papillary carcinoma invaded the capsule of the right lobe of the thyroid and metastasized to the cervical lymph nodes, warranting radioactive iodine therapy. However, considering the potential negative impact of radioactive iodine on preexisting lymphoma, the radioactive iodine therapy was postponed. In the low-risk group, medullary carcinoma in the left lobe of the thyroid requires long-term postoperative monitoring of serum calcitonin levels. Because the tumor is low-risk, no medication is required. This also suggests that in the case of coexistent tumors, it is essential to consider not only the treatment for each type of tumor but also the potential risks or conflicts associated with the treatments.

The treatment of SLL depends on the presentation of CLL. CLL or SLL is a low-grade lymphoid neoplasm characterized by small round B lymphocytes in the peripheral blood, bone marrow, and lymph nodes. The term SLL is reserved for CLL cases with tissue involvement.^[23]^ In the present case, SLL involved only the cervical lymph nodes without peripheral blood lymphocytosis. According to the treatment guidelines, patients with CLL/SLL should not receive treatment unless they exhibit the following: progressive bone marrow failure, splenomegaly, bulky lymph nodes (longest diameter > 10 cm), progressive lymphocytosis, symptomatic organ dysfunction caused by CLL/SLL, autoimmune hemolytic anemia, significant weight loss, severe fatigue, unexplained fever, night sweats.^[24]^ The patient in this case did not meet the above criteria and, following consultation with a hematologist, did not require treatment for CLL at the time. Six months after surgery, follow-up in the hematology department showed no lymphoma progression or recurrence.

Thus, when thyroid carcinoma and lymphoma coexist in the same patient, treatment strategies should be individualized, focusing on whether the tumor is at an advanced stage and condition. However, an ideal strategy entails the optimal treatment of both tumors.

The limitations of this study include the scarcity of relevant cases, which prevents a more in-depth exploration of the underlying pathogenesis. Additionally, the follow-up period is relatively short, though we plan to conduct long-term follow-up for this case.

## 4. Conclusion

Coexistence of PTC and MTC in the same patient is rare, but its incidence has increased over the past 30 years. While the coexistence of lymphoma and thyroid cancer is rare with only 1 case been reported in the literature, our study is the first to identify lymphomas in lymph nodes with cancer metastasis. This case highlights the importance of not only detecting cancer metastasis in lymph nodes but also considering the possibility of lymphoma in nodes showing structural and cellular abnormalities. Moreover, an effective system for distinguishing the origins of coexisting tumors is required, and effective therapeutic regimens for multiple tumors with few side effects need to be developed.

## Author contributions

**Data curation:** Zengfang Hao, Hanjing Cui, Haijun Dan.

**Formal analysis:** Zengfang Hao.

**Funding acquisition:** Zengfang Hao, Pengxin Zhao.

**Investigation:** Yuan Wang.

**Methodology:** Zengfang Hao, Yuan Wang, Lei Lou.

**Project administration:** Yuehong Li, Wenxin Wu.

**Resources:** Zengfang Hao, Pengxin Zhao.

**Software:** Zengfang Hao, Hengshu Wang.

**Supervision:** Pengxin Zhao.

**Visualization:** Hanjing Cui.

**Writing – original draft:** Zengfang Hao.

**Writing – review & editing:** Pengxin Zhao.
